# Diethyl 4-(4,5-dihydro­furan-2-yl)-3,5-di­methyl-1-phenyl-1,4-dihydro­pyrazine-2,6-dicarboxyl­ate

**DOI:** 10.1107/S1600536809013749

**Published:** 2009-04-25

**Authors:** Jing-Yu He, Zhi-Lin Tan, Hong Yan

**Affiliations:** a College of Life Science and Bioengineering, Beijing University of Technology, Beijing 100124, People’s Republic of China

## Abstract

In the title compound, C_22_H_26_N_2_O_5_, the central 1,4-dihydro­pyrazine ring adopts a boat conformation, while the benzene ring and the two disordered components of the furan ring are inclined at angles of 77.9 (5) and 61.9 (7)°. Three of the C atoms of the furan ring are disordered over two positions with occupancies of 0.655 (18) and 0.345 (18). In the crystal structure, weak inter­molecular C—H⋯O hydrogen bonds link the mol­ecules into chains propagating in [010].

## Related literature

For the biological properties of 1,4-dihydro­pyrazines, see: Goto *et al.* (1968 ▶); Teranishi & Goto (1990 ▶). For their biomedical applications, see: Brook *et al.* (1992 ▶); Sit *et al.* (2002 ▶). For the synthesis of 1,4-dihydro­pyrazines, see: Wolfbeis (1977 ▶); Chorvat & Rorig (1988 ▶); Rodrigues *et al.* (2004 ▶).
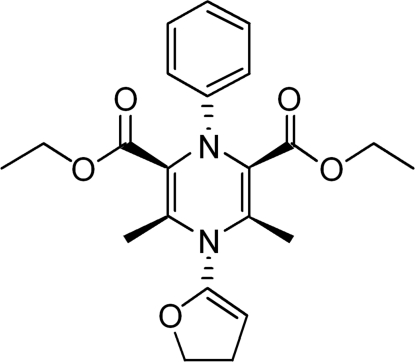


## Experimental

### 

#### Crystal data

C_22_H_26_N_2_O_5_
*M* *_r_* = 398.45
Triclinic, 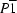

*a* = 10.069 (2) Å
*b* = 10.242 (2) Å
*c* = 12.519 (3) Å
α = 72.37 (3)°
β = 77.59 (3)°
γ = 63.76 (3)°
*V* = 1098.8 (4) Å^3^
*Z* = 2
Mo *K*α radiation
μ = 0.09 mm^−1^
*T* = 293 K
0.50 × 0.40 × 0.25 mm

#### Data collection

Rigaku R-AXIS RAPID IP diffractometer
Absorption correction: multi-scan (*ABSCOR*; Higashi, 1995 ▶) *T*_min_ = 0.958, *T*_max_ = 0.979
7345 measured reflections
3768 independent reflections
1555 reflections with *I* > 2σ(*I*)
*R*_int_ = 0.045

#### Refinement

*R*[*F* ^2^ > 2σ(*F* ^2^)] = 0.059
*wR*(*F* ^2^) = 0.182
*S* = 0.84
3768 reflections
291 parameters
52 restraints
H-atom parameters constrained
Δρ_max_ = 0.17 e Å^−3^
Δρ_min_ = −0.13 e Å^−3^

### 

Data collection: *RAPID-AUTO* (Rigaku, 2000 ▶); cell refinement: *RAPID-AUTO*; data reduction: *CrystalStructure* (Rigaku/MSC, 2000 ▶); program(s) used to solve structure: *SHELXS97* (Sheldrick, 2008 ▶); program(s) used to refine structure: *SHELXL97* (Sheldrick, 2008 ▶); molecular graphics: *SHELXTL* (Sheldrick, 2008 ▶); software used to prepare material for publication: *SHELXL97*.

## Supplementary Material

Crystal structure: contains datablocks I, global. DOI: 10.1107/S1600536809013749/hb2937sup1.cif

Structure factors: contains datablocks I. DOI: 10.1107/S1600536809013749/hb2937Isup2.hkl

Additional supplementary materials:  crystallographic information; 3D view; checkCIF report

## Figures and Tables

**Table 1:** Hydrogen-bond geometry (Å, °)

*D*—H⋯*A*	*D*—H	H⋯*A*	*D*⋯*A*	*D*—H⋯*A*
C12—H12*C*⋯O5^i^	0.96	2.67	3.618 (5)	169

Symmetry code: (i) 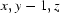.

**Fig. 1.**
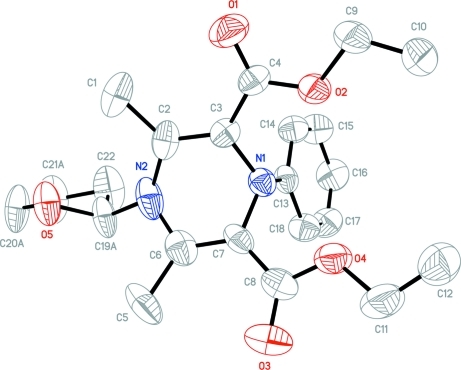
The molecular structure of (I) showing only the major disorder component of the furan ring. Displacement ellipsoids are drawn at the 30% probability level and H atoms have been omitted for clarity.

## supplementary crystallographic information

### Comment

The application of 1,4-dihydropyrazines in the field of biological agents and medicines has been widely investigated (Brook, *et al.*, 1992, Sit, *et al.*, 2002.), because 1,4-dihydropyrazine unit was found to be a component of the flavin coenzymes and several marine luciferins (Goto *et al.*, 1968; Teranishi & Goto, 1990). Although the synthesis of 1,4-dihydropyrazines has been studied for many years (Wolfbeis 1977; Chorvat & Rorig 1988; Rodrigues *et al.* 2004), their photochemical properties have not been paid much attention in the literature to date.

The photochemical stability of 2,6-diethoxycarbonyl-3,5-dimethyl-1-phenyl-1,4-dihydro- pyrazine (II) was investigated in a variety of conventional solvents such as benzene, THF, acetone, ethyl acetate, ethyl nitrile, n-hexane, ether, methanol and dichloromethane. In THF, the title compound (I), was obtained in a yield of *ca* 5% after irradiation for 8 h with a high-pressure Hg lamp. A similar transformation also occurred by irradiation with sunlight, ultraviolet, or other lower powered light sources. The present X-ray crystal structure analysis was undertaken, to study the stereochemistry and crystal packing of (I).

In (I) (Fig. 1), the 1,4-dihydropyrazine ring (N1/C3/C2/N2/C6/C7) adopts a boat conformation: atoms C2, C3, C6 and C7 are coplanar, with atoms N1 and N2 deviating from this plane by 0.517 (4) and 0.362 (5) Å, respectively. The dihedral angle between the phenyl ring and C2/C3/C6/C7 plane is 70.46 (18)°. with those between the phenyl ring and the two disorder components of the furan ring are 77.9 (5)° and 61.9 (7)° respectively. In the crystal structure, weak intermolecular C—H···O hydrogen bonds (Table 1) link the molecules into chains propagated along *b* axis.

### Experimental

Diethyl 3,5-dimethyl-1-phenyl-1,4-dihydropyrazine-2,6-dicarboxylate, (330 mg, 1 mmol) was dissolved in dry furan (30 ml) and poured into the photolysis unit. The solution was irradiated with a 500 W Hg lamp. The reaction was monitored by TLC. After 8 h, the solvent was removed *in vacuo* and the crude sample was purified on a silica-gel column using an n-hexane/ethyl acetate (20:1 *v*/*v*) as eluant. Colourless blocks of (I) were obtained by slow evaporation of a n-hexane / ethyl acetate solution (3:1 *v*/*v*) in a yield of 5.2% (21 mg; m.p. 421–423 K).

### Refinement

All H-atoms were positioned geometrically (C—H = 0.93–0.96Å) and refined as riding with *U*_iso_(H) = 1.2*U*_eq_(C) or 1.5U_eq_(methyl C).   Some cardon atoms in the furan ring refined with very anisotropic displacemet factors, indicating positional disorder. In the chosen disorder model, atoms C19, C20 and C21 were disordered over two positions with refined occupancies of 0.655 (18) and 0.345 (18).  However, high atomic displacement parameters for these and their neighbouring atoms indicates that additional unresolved disorder may also be present.

### Crystal data

**Table:**

C_22_H_26_N_2_O_5_	*Z* = 2
*M**_r_* = 398.45	*F*(000) = 424
Triclinic, *P*1	*D*_x_ = 1.204 Mg m^−^^3^
Hall symbol: -P 1	Mo *K*α radiation, λ = 0.71073 Å
*a* = 10.069 (2) Å	Cell parameters from 7345 reflections
*b* = 10.242 (2) Å	θ = 2.3–25.0°
*c* = 12.519 (3) Å	µ = 0.09 mm^−^^1^
α = 72.37 (3)°	*T* = 293 K
β = 77.59 (3)°	Block, colourless
γ = 63.76 (3)°	0.50 × 0.40 × 0.25 mm
*V* = 1098.8 (4) Å^3^	

### Data collection

**Table:**

Rigaku R-AXIS RAPID IP diffractometer	3768 independent reflections
Radiation source: fine-focus sealed tube	1555 reflections with *I* > 2σ(*I*)
graphite	*R*_int_ = 0.045
Detector resolution: 10.00 pixels mm^-1^	θ_max_ = 25.0°, θ_min_ = 2.3°
ω scans	*h* = −11→10
Absorption correction: multi-scan (*ABSCOR*; Higashi, 1995)	*k* = −12→12
*T*_min_ = 0.958, *T*_max_ = 0.979	*l* = −14→13
7345 measured reflections	

### Refinement

**Table:**

Refinement on *F*^2^	Secondary atom site location: difference Fourier map
Least-squares matrix: full	Hydrogen site location: inferred from neighbouring sites
*R*[*F*^2^ > 2σ(*F*^2^)] = 0.059	H-atom parameters constrained
*wR*(*F*^2^) = 0.182	*w* = 1/[σ^2^(*F*_o_^2^) + (0.102*P*)^2^] where *P* = (*F*_o_^2^ + 2*F*_c_^2^)/3
*S* = 0.84	(Δ/σ)_max_ = 0.005
3768 reflections	Δρ_max_ = 0.17 e Å^−^^3^
291 parameters	Δρ_min_ = −0.13 e Å^−^^3^
52 restraints	Extinction correction: *SHELXL97* (Sheldrick, 2008), Fc^*^=kFc[1+0.001xFc^2^λ^3^/sin(2θ)]^-1/4^
Primary atom site location: structure-invariant direct methods	Extinction coefficient: 0.034 (5)

### Special details

**Table:**

Geometry. All e.s.d.'s (except the e.s.d. in the dihedral angle between two l.s. planes) are estimated using the full covariance matrix. The cell e.s.d.'s are taken into account individually in the estimation of e.s.d.'s in distances, angles and torsion angles; correlations between e.s.d.'s in cell parameters are only used when they are defined by crystal symmetry. An approximate (isotropic) treatment of cell e.s.d.'s is used for estimating e.s.d.'s involving l.s. planes.
Refinement. Refinement of *F*^2^ against ALL reflections. The weighted *R*-factor *wR* and goodness of fit *S* are based on *F*^2^, conventional *R*-factors *R* are based on *F*, with *F* set to zero for negative *F*^2^. The threshold expression of *F*^2^ > σ(*F*^2^) is used only for calculating *R*-factors(gt) *etc*. and is not relevant to the choice of reflections for refinement. *R*-factors based on *F*^2^ are statistically about twice as large as those based on *F*, and *R*- factors based on ALL data will be even larger.

### Fractional atomic coordinates and isotropic or equivalent isotropic displacement parameters (Å^2^)

**Table:**

	*x*	*y*	*z*	*U*_iso_*/*U*_eq_	Occ. (<1)
O1	0.6304 (3)	0.7169 (3)	0.1028 (2)	0.1226 (10)	
O2	0.5222 (2)	0.5632 (3)	0.19260 (19)	0.0867 (7)	
O3	0.1854 (3)	0.7014 (4)	0.5774 (2)	0.1395 (12)	
O4	0.2987 (3)	0.5488 (3)	0.46179 (19)	0.1043 (9)	
N1	0.2832 (3)	0.7643 (3)	0.2761 (2)	0.0696 (7)	
N2	0.2680 (3)	1.0189 (3)	0.2870 (4)	0.1035 (11)	
C1	0.4963 (4)	1.0117 (4)	0.1529 (3)	0.1177 (15)	
H1A	0.4623	1.1095	0.1661	0.177*	
H1B	0.5047	1.0198	0.0735	0.177*	
H1C	0.5917	0.9491	0.1810	0.177*	
C2	0.3879 (4)	0.9443 (4)	0.2120 (3)	0.0866 (11)	
C3	0.4005 (3)	0.8073 (4)	0.2125 (3)	0.0699 (9)	
C4	0.5280 (4)	0.6958 (4)	0.1640 (3)	0.0765 (9)	
C5	0.1917 (4)	0.9956 (5)	0.4888 (4)	0.1324 (17)	
H5A	0.1863	1.0960	0.4653	0.199*	
H5B	0.2671	0.9352	0.5399	0.199*	
H5C	0.0974	0.9967	0.5258	0.199*	
C6	0.2298 (4)	0.9315 (5)	0.3880 (4)	0.0951 (12)	
C7	0.2464 (3)	0.7984 (4)	0.3838 (3)	0.0760 (9)	
C8	0.2383 (4)	0.6822 (5)	0.4836 (4)	0.0944 (11)	
C9	0.6449 (4)	0.4402 (4)	0.1523 (3)	0.1075 (13)	
H9A	0.6539	0.4641	0.0707	0.129*	
H9B	0.7370	0.4254	0.1762	0.129*	
C10	0.6198 (5)	0.3060 (5)	0.1958 (4)	0.1389 (18)	
H10A	0.7012	0.2253	0.1686	0.208*	
H10B	0.5290	0.3207	0.1714	0.208*	
H10C	0.6124	0.2818	0.2766	0.208*	
C11	0.3014 (6)	0.4218 (5)	0.5561 (4)	0.1469 (19)	
H11A	0.3477	0.4207	0.6169	0.176*	
H11B	0.2005	0.4318	0.5837	0.176*	
C12	0.3823 (5)	0.2857 (6)	0.5208 (4)	0.1332 (16)	
H12A	0.3808	0.2031	0.5824	0.200*	
H12B	0.4833	0.2739	0.4966	0.200*	
H12C	0.3375	0.2880	0.4595	0.200*	
C13	0.1673 (3)	0.7858 (3)	0.2185 (3)	0.0658 (8)	
C14	0.1859 (3)	0.7952 (4)	0.1046 (3)	0.0800 (10)	
H14	0.2763	0.7905	0.0642	0.096*	
C15	0.0733 (4)	0.8114 (4)	0.0497 (3)	0.1003 (12)	
H15	0.0873	0.8206	−0.0276	0.120*	
C16	−0.0604 (4)	0.8142 (4)	0.1082 (4)	0.1045 (12)	
H16	−0.1357	0.8220	0.0716	0.125*	
C17	−0.0797 (4)	0.8053 (4)	0.2212 (4)	0.0980 (12)	
H17	−0.1702	0.8096	0.2612	0.118*	
C18	0.0310 (3)	0.7903 (4)	0.2771 (3)	0.0848 (10)	
H18	0.0155	0.7831	0.3542	0.102*	
O5	0.2634 (3)	1.2572 (3)	0.2789 (2)	0.1113 (9)	
C22	0.0984 (5)	1.2576 (5)	0.1768 (4)	0.1378 (18)	
H22	0.0796	1.2188	0.1262	0.165*	
C19A	0.1706 (10)	1.1824 (7)	0.2786 (9)	0.098 (3)	0.655 (18)
C20A	0.1744 (15)	1.4006 (12)	0.2440 (12)	0.133 (4)	0.655 (18)
H20A	0.2337	1.4551	0.1997	0.160*	0.655 (18)
H20B	0.1250	1.4425	0.3089	0.160*	0.655 (18)
C21A	0.0607 (17)	1.4213 (11)	0.1753 (14)	0.152 (5)	0.655 (18)
H21A	0.0713	1.4796	0.0994	0.182*	0.655 (18)
H21B	−0.0392	1.4690	0.2094	0.182*	0.655 (18)
C19B	0.2390 (19)	1.1784 (12)	0.2077 (18)	0.107 (6)	0.345 (18)
C20B	0.206 (4)	1.427 (3)	0.209 (3)	0.208 (17)	0.345 (18)
H20C	0.1396	1.4977	0.2540	0.250*	0.345 (18)
H20D	0.2851	1.4567	0.1676	0.250*	0.345 (18)
C21B	0.127 (3)	1.400 (2)	0.138 (2)	0.163 (12)	0.345 (18)
H21C	0.1837	1.3997	0.0645	0.196*	0.345 (18)
H21D	0.0321	1.4850	0.1274	0.196*	0.345 (18)

### Atomic displacement parameters (Å^2^)

**Table:**

	*U*^11^	*U*^22^	*U*^33^	*U*^12^	*U*^13^	*U*^23^
O1	0.0867 (18)	0.118 (2)	0.146 (2)	−0.0465 (16)	0.0294 (17)	−0.0275 (18)
O2	0.0683 (14)	0.0740 (16)	0.1007 (17)	−0.0191 (12)	0.0113 (12)	−0.0256 (13)
O3	0.131 (2)	0.176 (3)	0.099 (2)	−0.048 (2)	0.0251 (18)	−0.063 (2)
O4	0.119 (2)	0.097 (2)	0.0760 (16)	−0.0355 (16)	0.0076 (14)	−0.0158 (15)
N1	0.0622 (16)	0.0669 (16)	0.0786 (18)	−0.0212 (12)	−0.0053 (14)	−0.0247 (14)
N2	0.081 (2)	0.063 (2)	0.171 (4)	−0.0093 (17)	−0.037 (2)	−0.048 (2)
C1	0.130 (3)	0.105 (3)	0.134 (3)	−0.076 (3)	−0.047 (3)	0.019 (3)
C2	0.091 (3)	0.064 (2)	0.108 (3)	−0.034 (2)	−0.045 (2)	0.005 (2)
C3	0.0558 (19)	0.069 (2)	0.075 (2)	−0.0206 (16)	−0.0101 (16)	−0.0075 (17)
C4	0.064 (2)	0.082 (3)	0.078 (2)	−0.0333 (19)	−0.0047 (18)	−0.008 (2)
C5	0.106 (3)	0.143 (4)	0.177 (4)	−0.029 (3)	−0.001 (3)	−0.118 (4)
C6	0.068 (2)	0.087 (3)	0.127 (4)	−0.017 (2)	−0.011 (2)	−0.044 (3)
C7	0.059 (2)	0.078 (2)	0.092 (3)	−0.0144 (17)	−0.0012 (17)	−0.047 (2)
C8	0.082 (3)	0.100 (3)	0.092 (3)	−0.020 (2)	−0.003 (2)	−0.040 (3)
C9	0.086 (3)	0.092 (3)	0.119 (3)	−0.018 (2)	0.018 (2)	−0.038 (2)
C10	0.133 (4)	0.095 (3)	0.172 (4)	−0.045 (3)	0.042 (3)	−0.051 (3)
C11	0.189 (5)	0.121 (4)	0.089 (3)	−0.057 (4)	0.021 (3)	0.000 (3)
C12	0.153 (4)	0.125 (4)	0.113 (4)	−0.059 (3)	−0.020 (3)	−0.009 (3)
C13	0.060 (2)	0.0530 (18)	0.083 (2)	−0.0165 (14)	−0.0107 (17)	−0.0202 (16)
C14	0.063 (2)	0.088 (2)	0.081 (3)	−0.0254 (17)	−0.0066 (18)	−0.0182 (19)
C15	0.083 (3)	0.124 (3)	0.086 (3)	−0.034 (2)	−0.022 (2)	−0.018 (2)
C16	0.073 (3)	0.118 (3)	0.120 (3)	−0.032 (2)	−0.023 (2)	−0.024 (3)
C17	0.062 (2)	0.111 (3)	0.115 (3)	−0.036 (2)	−0.001 (2)	−0.023 (3)
C18	0.060 (2)	0.101 (3)	0.090 (2)	−0.0280 (18)	−0.0035 (19)	−0.029 (2)
O5	0.1092 (18)	0.0695 (18)	0.166 (3)	−0.0273 (15)	−0.0492 (17)	−0.0316 (17)
C22	0.164 (5)	0.087 (3)	0.166 (4)	−0.034 (3)	−0.097 (4)	−0.006 (3)
C19A	0.088 (5)	0.053 (4)	0.160 (8)	−0.010 (3)	−0.044 (5)	−0.042 (4)
C20A	0.155 (9)	0.044 (5)	0.207 (10)	−0.045 (6)	−0.056 (8)	−0.004 (7)
C21A	0.141 (9)	0.093 (7)	0.218 (12)	−0.029 (6)	−0.094 (9)	−0.008 (7)
C19B	0.082 (10)	0.069 (8)	0.178 (15)	−0.004 (6)	−0.049 (9)	−0.054 (9)
C20B	0.152 (17)	0.117 (19)	0.32 (3)	−0.097 (14)	−0.059 (16)	0.092 (17)
C21B	0.19 (3)	0.073 (12)	0.184 (18)	−0.042 (14)	0.037 (17)	−0.033 (11)

### Geometric parameters (Å, °)

**Table:**

O1—C4	1.207 (3)	C12—H12A	0.9600
O2—C4	1.320 (4)	C12—H12B	0.9600
O2—C9	1.452 (4)	C12—H12C	0.9600
O3—C8	1.216 (4)	C13—C14	1.376 (4)
O4—C8	1.316 (4)	C13—C18	1.398 (4)
O4—C11	1.462 (4)	C14—C15	1.373 (4)
N1—C13	1.404 (4)	C14—H14	0.9300
N1—C7	1.426 (4)	C15—C16	1.380 (5)
N1—C3	1.437 (4)	C15—H15	0.9300
N2—C6	1.399 (5)	C16—C17	1.367 (5)
N2—C2	1.433 (4)	C16—H16	0.9300
N2—C19A	1.504 (7)	C17—C18	1.368 (5)
N2—C19B	1.568 (15)	C17—H17	0.9300
C1—C2	1.492 (5)	C18—H18	0.9300
C1—H1A	0.9600	O5—C20A	1.329 (12)
C1—H1B	0.9600	O5—C19A	1.448 (8)
C1—H1C	0.9600	O5—C19B	1.488 (13)
C2—C3	1.350 (4)	O5—C20B	1.59 (2)
C3—C4	1.453 (4)	C22—C19B	1.357 (12)
C5—C6	1.498 (5)	C22—C19A	1.433 (8)
C5—H5A	0.9600	C22—C21B	1.530 (17)
C5—H5B	0.9600	C22—C21A	1.541 (10)
C5—H5C	0.9600	C22—H22	0.9300
C6—C7	1.314 (5)	C20A—C21A	1.480 (11)
C7—C8	1.458 (5)	C20A—H20A	0.9700
C9—C10	1.430 (5)	C20A—H20B	0.9700
C9—H9A	0.9700	C21A—H21A	0.9700
C9—H9B	0.9700	C21A—H21B	0.9700
C10—H10A	0.9600	C20B—C21B	1.460 (19)
C10—H10B	0.9600	C20B—H20C	0.9700
C10—H10C	0.9600	C20B—H20D	0.9700
C11—C12	1.420 (5)	C21B—H21C	0.9700
C11—H11A	0.9700	C21B—H21D	0.9700
C11—H11B	0.9700		
			
C4—O2—C9	118.7 (3)	C14—C13—C18	118.0 (3)
C8—O4—C11	117.1 (3)	C14—C13—N1	121.6 (3)
C13—N1—C7	118.2 (2)	C18—C13—N1	120.4 (3)
C13—N1—C3	118.2 (3)	C15—C14—C13	121.1 (3)
C7—N1—C3	111.3 (3)	C15—C14—H14	119.5
C6—N2—C2	118.2 (3)	C13—C14—H14	119.5
C6—N2—C19A	110.6 (6)	C14—C15—C16	120.7 (4)
C2—N2—C19A	131.2 (6)	C14—C15—H15	119.7
C6—N2—C19B	148.5 (9)	C16—C15—H15	119.7
C2—N2—C19B	93.3 (8)	C17—C16—C15	118.4 (4)
C19A—N2—C19B	37.9 (5)	C17—C16—H16	120.8
C2—C1—H1A	109.5	C15—C16—H16	120.8
C2—C1—H1B	109.5	C16—C17—C18	121.6 (3)
H1A—C1—H1B	109.5	C16—C17—H17	119.2
C2—C1—H1C	109.5	C18—C17—H17	119.2
H1A—C1—H1C	109.5	C17—C18—C13	120.2 (3)
H1B—C1—H1C	109.5	C17—C18—H18	119.9
C3—C2—N2	114.5 (3)	C13—C18—H18	119.9
C3—C2—C1	125.7 (4)	C20A—O5—C19A	102.0 (6)
N2—C2—C1	119.4 (4)	C20A—O5—C19B	104.9 (6)
C2—C3—N1	116.4 (3)	C19A—O5—C19B	39.8 (6)
C2—C3—C4	125.7 (3)	C20A—O5—C20B	19.4 (15)
N1—C3—C4	117.7 (3)	C19A—O5—C20B	113.8 (11)
O1—C4—O2	121.2 (3)	C19B—O5—C20B	103.7 (12)
O1—C4—C3	125.7 (3)	C19B—C22—C19A	41.9 (7)
O2—C4—C3	113.1 (3)	C19B—C22—C21B	89.5 (15)
C6—C5—H5A	109.5	C19A—C22—C21B	102.7 (12)
C6—C5—H5B	109.5	C19B—C22—C21A	105.4 (7)
H5A—C5—H5B	109.5	C19A—C22—C21A	99.8 (6)
C6—C5—H5C	109.5	C21B—C22—C21A	27.0 (11)
H5A—C5—H5C	109.5	C19B—C22—H22	111.8
H5B—C5—H5C	109.5	C19A—C22—H22	130.1
C7—C6—N2	115.8 (4)	C21B—C22—H22	121.4
C7—C6—C5	128.6 (5)	C21A—C22—H22	130.1
N2—C6—C5	115.3 (4)	C22—C19A—O5	106.0 (6)
C6—C7—N1	117.6 (4)	C22—C19A—N2	114.1 (6)
C6—C7—C8	123.3 (4)	O5—C19A—N2	108.2 (5)
N1—C7—C8	118.9 (3)	O5—C20A—C21A	112.4 (8)
O3—C8—O4	122.4 (4)	O5—C20A—H20A	109.1
O3—C8—C7	125.8 (4)	C21A—C20A—H20A	109.1
O4—C8—C7	111.8 (3)	O5—C20A—H20B	109.1
C10—C9—O2	110.0 (3)	C21A—C20A—H20B	109.1
C10—C9—H9A	109.7	H20A—C20A—H20B	107.9
O2—C9—H9A	109.7	C20A—C21A—C22	101.2 (7)
C10—C9—H9B	109.7	C20A—C21A—H21A	111.5
O2—C9—H9B	109.7	C22—C21A—H21A	111.5
H9A—C9—H9B	108.2	C20A—C21A—H21B	111.5
C9—C10—H10A	109.5	C22—C21A—H21B	111.5
C9—C10—H10B	109.5	H21A—C21A—H21B	109.4
H10A—C10—H10B	109.5	C22—C19B—O5	107.9 (10)
C9—C10—H10C	109.5	C22—C19B—N2	114.6 (12)
H10A—C10—H10C	109.5	O5—C19B—N2	103.0 (11)
H10B—C10—H10C	109.5	C21B—C20B—O5	92.0 (15)
C12—C11—O4	110.0 (4)	C21B—C20B—H20C	113.3
C12—C11—H11A	109.7	O5—C20B—H20C	113.3
O4—C11—H11A	109.7	C21B—C20B—H20D	113.3
C12—C11—H11B	109.7	O5—C20B—H20D	113.3
O4—C11—H11B	109.7	H20C—C20B—H20D	110.6
H11A—C11—H11B	108.2	C20B—C21B—C22	118.7 (18)
C11—C12—H12A	109.5	C20B—C21B—H21C	107.6
C11—C12—H12B	109.5	C22—C21B—H21C	107.6
H12A—C12—H12B	109.5	C20B—C21B—H21D	107.6
C11—C12—H12C	109.5	C22—C21B—H21D	107.6
H12A—C12—H12C	109.5	H21C—C21B—H21D	107.1
H12B—C12—H12C	109.5		
			
C6—N2—C2—C3	33.1 (4)	C14—C13—C18—C17	−0.7 (5)
C19A—N2—C2—C3	−147.6 (4)	N1—C13—C18—C17	−177.4 (3)
C19B—N2—C2—C3	−147.0 (5)	C19B—C22—C19A—O5	61.0 (10)
C6—N2—C2—C1	−139.4 (3)	C21B—C22—C19A—O5	−13.7 (15)
C19A—N2—C2—C1	39.8 (6)	C21A—C22—C19A—O5	−41.1 (12)
C19B—N2—C2—C1	40.5 (5)	C19B—C22—C19A—N2	−58.0 (10)
N2—C2—C3—N1	7.5 (4)	C21B—C22—C19A—N2	−132.6 (11)
C1—C2—C3—N1	179.5 (3)	C21A—C22—C19A—N2	−160.1 (7)
N2—C2—C3—C4	−166.2 (3)	C20A—O5—C19A—C22	42.6 (11)
C1—C2—C3—C4	5.8 (5)	C19B—O5—C19A—C22	−56.4 (9)
C13—N1—C3—C2	96.3 (3)	C20B—O5—C19A—C22	26.4 (16)
C7—N1—C3—C2	−45.5 (4)	C20A—O5—C19A—N2	165.4 (7)
C13—N1—C3—C4	−89.5 (3)	C19B—O5—C19A—N2	66.4 (9)
C7—N1—C3—C4	128.7 (3)	C20B—O5—C19A—N2	149.2 (13)
C9—O2—C4—O1	2.2 (5)	C6—N2—C19A—C22	−126.8 (7)
C9—O2—C4—C3	−178.4 (3)	C2—N2—C19A—C22	53.9 (9)
C2—C3—C4—O1	−11.4 (5)	C19B—N2—C19A—C22	52.9 (9)
N1—C3—C4—O1	175.0 (3)	C6—N2—C19A—O5	115.4 (6)
C2—C3—C4—O2	169.1 (3)	C2—N2—C19A—O5	−63.9 (8)
N1—C3—C4—O2	−4.5 (4)	C19B—N2—C19A—O5	−64.9 (10)
C2—N2—C6—C7	−33.5 (5)	C19A—O5—C20A—C21A	−25.3 (13)
C19A—N2—C6—C7	147.1 (4)	C19B—O5—C20A—C21A	15.6 (16)
C19B—N2—C6—C7	146.8 (9)	C20B—O5—C20A—C21A	104 (4)
C2—N2—C6—C5	140.0 (3)	O5—C20A—C21A—C22	0.7 (14)
C19A—N2—C6—C5	−39.4 (5)	C19B—C22—C21A—C20A	−18.3 (17)
C19B—N2—C6—C5	−39.7 (10)	C19A—C22—C21A—C20A	24.3 (13)
N2—C6—C7—N1	−7.5 (4)	C21B—C22—C21A—C20A	−74 (3)
C5—C6—C7—N1	−180.0 (3)	C19A—C22—C19B—O5	−59.3 (12)
N2—C6—C7—C8	168.5 (3)	C21B—C22—C19B—O5	50.5 (17)
C5—C6—C7—C8	−3.9 (6)	C21A—C22—C19B—O5	28.3 (19)
C13—N1—C7—C6	−95.6 (4)	C19A—C22—C19B—N2	54.7 (13)
C3—N1—C7—C6	46.2 (4)	C21B—C22—C19B—N2	164.5 (13)
C13—N1—C7—C8	88.2 (3)	C21A—C22—C19B—N2	142.4 (9)
C3—N1—C7—C8	−130.0 (3)	C20A—O5—C19B—C22	−28.3 (19)
C11—O4—C8—O3	−0.6 (6)	C19A—O5—C19B—C22	62.6 (12)
C11—O4—C8—C7	178.3 (3)	C20B—O5—C19B—C22	−48 (2)
C6—C7—C8—O3	16.8 (6)	C20A—O5—C19B—N2	−149.9 (8)
N1—C7—C8—O3	−167.2 (3)	C19A—O5—C19B—N2	−58.9 (12)
C6—C7—C8—O4	−162.0 (3)	C20B—O5—C19B—N2	−169.8 (13)
N1—C7—C8—O4	14.0 (4)	C6—N2—C19B—C22	−57.1 (17)
C4—O2—C9—C10	177.9 (3)	C2—N2—C19B—C22	123.1 (12)
C8—O4—C11—C12	−174.4 (4)	C19A—N2—C19B—C22	−57.7 (13)
C7—N1—C13—C14	162.1 (3)	C6—N2—C19B—O5	59.8 (14)
C3—N1—C13—C14	23.0 (4)	C2—N2—C19B—O5	−120.0 (10)
C7—N1—C13—C18	−21.2 (4)	C19A—N2—C19B—O5	59.2 (11)
C3—N1—C13—C18	−160.4 (3)	C20A—O5—C20B—C21B	−81 (4)
C18—C13—C14—C15	1.2 (5)	C19A—O5—C20B—C21B	−25 (2)
N1—C13—C14—C15	177.9 (3)	C19B—O5—C20B—C21B	16 (2)
C13—C14—C15—C16	−1.9 (5)	O5—C20B—C21B—C22	16 (3)
C14—C15—C16—C17	2.1 (6)	C19B—C22—C21B—C20B	−43 (3)
C15—C16—C17—C18	−1.6 (6)	C19A—C22—C21B—C20B	−3(3)
C16—C17—C18—C13	0.9 (5)	C21A—C22—C21B—C20B	84 (3)

### Hydrogen-bond geometry (Å, °)

**Table:**

*D*—H···*A*	*D*—H	H···*A*	*D*···*A*	*D*—H···*A*
C12—H12C···O5^i^	0.96	2.67	3.618 (5)	169

Symmetry codes: (i) *x*, *y*−1, *z*.
